# The Effect of Resistance Training on Motor Unit Firing Properties: A Systematic Review and Meta-Analysis

**DOI:** 10.3389/fphys.2022.817631

**Published:** 2022-02-28

**Authors:** Edith Elgueta-Cancino, Ethan Evans, Eduardo Martinez-Valdes, Deborah Falla

**Affiliations:** Centre of Precision Rehabilitation for Spinal Pain (CPR Spine), School of Sport, Exercise and Rehabilitation Sciences, University of Birmingham, Birmingham, United Kingdom

**Keywords:** resistance training, strength training, motor unit discharge rate, motor unit discharge rate variability, motor unit recruitment threshold, motor unit (MU)

## Abstract

While neural changes are thought to be responsible for early increases in strength following resistance training (RT), the exact changes in motor unit (MU) firing properties remain unclear. This review aims to synthesize the available evidence on the effect of RT on MU firing properties. MEDLINE (OVID interface), EMBASE (OVID interface), Web of Science (all databases), Cochrane Library, EBSCO CINAHL Plus, PubMed, and EBSCO SportDiscus were searched from inception until June 2021. Randomized controlled trials and non-randomized studies of interventions that compared RT to no intervention (control) were included. Two reviewers independently extracted data from each trial, assessed the risk of bias and rated the cumulative quality of evidence. Motor unit discharge rate (MUDR), motor unit recruitment threshold (MURT), motor unit discharge rate variability (MUDRV), MU discharge rate at recruitment vs. recruitment threshold relationship, and MU discharge rate vs. recruitment threshold relationship were assessed. Seven trials including 167 participants met the inclusion criteria. Meta-analysis (four studies) revealed that MUDR did not change significantly (*P* = 0.43), but with considerable heterogeneity likely to be present (*I*^2^ = 91). Low to moderate evidence supports changes in MUDRV, MUDR at recruitment vs. recruitment threshold relationship, and the MUDR vs. recruitment threshold relationship. Overall, this systematic review revealed that there is a lack of high-quality evidence for the effect of RT on MU firing properties. Heterogeneity across studies undermines the quality of the evidence for multiple outcomes and affects the conclusions that can be drawn.

## Introduction

Resistance training (RT) involves resisted movements with the overall goal to increase an individual’s strength. Numerous muscular and neurophysiological effects can be seen in the target muscles, such as, an increase in muscle volume and physiological cross-sectional area (Maden-Wilkinson et al., 2020) and neural adaptations (Gabriel et al., 2001, 2006; Škarabot et al., 2019; Aagaard et al., 2020; Hortobágyi et al., 2021; Pearcey et al., 2021). The increase in strength in response to RT occurs before morphological changes such as hypertrophy (i.e., muscle size increase) are measurable (Akima et al., 1999). A recent systematic review suggests that plastic changes at different levels of central nervous system such as, decreased activity of inhibitory networks in the primary motor cortex (Kidgell et al., 2017), increased corticospinal axon excitability at the spinal level (Mason et al., 2019) and changes in motor unit activation (Siddique et al., 2020) are responsible for early changes in strength output (Siddique et al., 2020). Additionally, skeletal muscle protein adaptations have been shown to occur within the first 2–4 weeks of resistance training (Staron et al., 1994) as recently discussed by Pearcey et al. (2021).

There is a growing body of research investigating changes in motor unit firing properties following RT. Amongst others, the most commonly investigated measures are: motor unit recruitment threshold (MURT), discharge rate (MUDR), and discharge rate variability (MUDRV) (Vila-Chã et al., 2010; Vila-Chã and Falla, 2016). For example, Del Vecchio et al. (2019a) reported a significant increase in MUDR and a significant decrease in MURT following 4-weeks of isometric RT of the tibialis anterior in young healthy adults. However, others have shown only early increases in MUDR with no changes after weeks of training or no change across the whole RT intervention. For instance, Patten et al. (2001), despite an initial significant increase in MUDR after 2 days of isometric RT of the digiti minimi, found no significant increase in MUDR after 42 days. Further, Pucci et al. (2006) found no significant change in MUDR after 3-weeks of isometric RT. These diverse findings make changes in motor unit firing properties following a regimen of RT unclear, and no systematic review has evaluated the overall evidence of changes in motor unit firing properties in response to RT in order to synthesize the available evidence and draw conclusions from the available studies. Thus, this systematic review aims to synthesize current evidence on the effect of RT on motor unit firing properties in order to determine the direction and strength of evidence.

## Methods

The reporting of this review follows the Preferred Reporting Items for Systematic Reviews and Meta-Analysis Protocols (PRISMA-P) (Page et al., 2021). The review was prospectively registered with PROSPERO (CRD42021236376) on the 11th of February 2021.

### Eligibility Criteria

The eligibility criteria were informed by the PICOS framework [P: Population; I: Intervention; C: Comparator; O: Outcome(s); S: Study design] (McKenzie et al., 2019) and are presented in Table 1.

**TABLE 1:** Eligibility criteria.

PICOS	Inclusion criteria	Exclusion criteria
Population	• Female and male • 18–65 years old • Healthy adults	• Current or history of musculoskeletal, neurological, or metabolic disorders and/or any participants currently experiencing pain.
Intervention	**Resistance training:**	• Spinal, pelvic musculature
	• Frequency: a minimum of 2 non-consecutive days per week • Resisted or weighted exercises involving the targeted muscle for studying	• Any muscle wholly located proximal to the shoulder and hip joints, unless these muscles were trained as part of a training program and were not the muscle being studied.
	• Multiple sets of 8–15 repetitions (depending on age), as per the American College for Sports and Medicine (ACSM) guidelines (Liguori and Medicine, 2020).	
	• The load must have been 60–85% of the participants one-repetition maximum (1 RM) or maximum voluntary contraction force (Gabriel et al., 2006).	
	• Duration: a minimum of 2 weeks	
	• Target: Upper or lower limb musculature only.	
Comparator	• Group(s) completing RT to a control group, or other groups not completing RT (placebo or group completing other forms of training, for example, aerobic training). • Studies comparing one of the outcome measures amongst other unrelated measures.	• Prospective cohort studies without comparison to a control or other reference group were excluded. • Any trial that combined RT with any other form of training (for example, aerobic training) was excluded.
Outcome measures	• Any trials measuring either MURT, MUDR, or MUDRV, following a course of RT. • Trials reporting these outcomes in linear relationships (for example, MUDR vs. MURT relationship). • The data must have been collected using surface or intramuscular electromyography (EMG).	• Any studies which were secondary analyses of primary published data were excluded unless the secondary reports included outcome measures not reported in the original study (for example, MUDR was reported in the first publication and MUDRV in the second publication).
	• Assessment data collected at any time point during training or upon completion of training	
Study design	• Randomized control trials (RCT).	• Systematic reviews and conference abstracts.
	• Non-randomized studies of interventions (NRSI).	• Articles not written in English.

### Search Strategy and Data Sources

The following databases were searched from the inception of each database until the 25th of June 2021: MEDLINE (OVID interface), EMBASE (OVID interface), Web of Science (all databases), Cochrane Library, EBSCO CINAHL Plus, PubMed, and EBSCO SportDiscus.

A MEDLINE search strategy was first planned and modified accordingly for the other databases (Supplementary Appendix 1). The search strategy combined terms relating to RT, MUDR, MURT, and MUDRV (Supplementary Appendix 2).

### Study Selection

**FIGURE 1:**
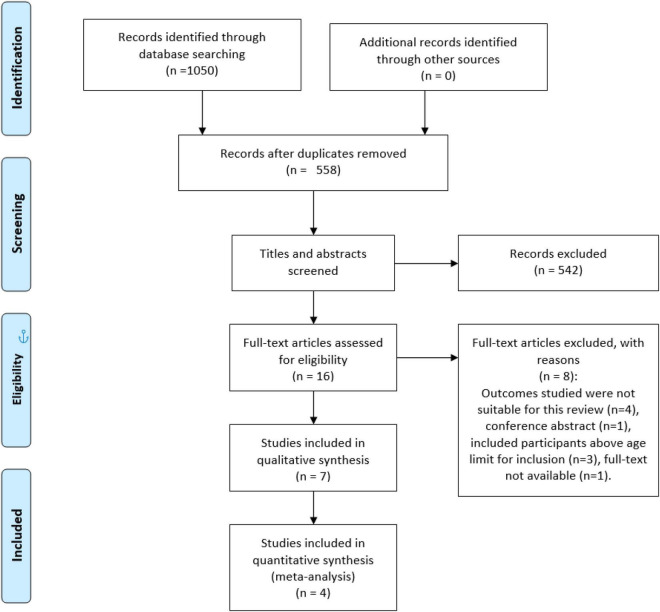
PRISMA flow diagram.

All results were managed with Clarivate Analytics Endnote (Version 20) software. Two reviewers (EE/EC) screened the title and abstracts of articles for inclusion following the eligibility criteria. When a study was classified as eligible the full text was screened to ensure eligibility (Cooper, 2015). If a text was rated as unsure or was disputed between the two reviewers, the text was discussed. In the event of a disagreement between the two reviewers, a third reviewer (DF) adjudicated the eligibility of the text. The number of included/excluded studies is presented with the PRISMA flow diagram with reasons for exclusions (Figure 1; Moher et al., 2010).

### Data Extraction

A data extraction form was created based on the Cochrane data collection form (Higgins et al., 2021). The form was tested on two articles to allow reviewers to practice and make any necessary alterations. Both reviewers independently extracted information from the studies meeting the inclusion criteria, with any discrepancies mediated by the third reviewer (DF).

### Risk of Bias in Individual Studies and Quality of the Evidence

The revised Cochrane risk of bias tool for randomized trials (RoB-2) (Higgins et al., 2021) was used independently by two reviewers (EE/EC) to assess the risk of bias of each of the included randomized controlled trials (RCTs). For non-randomized studies on intervention effects (NRSIs), the Risk Of Bias In Non-randomized Studies of Interventions (ROBINS-I) tool was used to assess the risk of bias.

Grading of Recommendations Assessment, Development and Evaluation (GRADE), was used to assess and present the overall certainty of evidence, following guidance from the GRADE handbook (Schünemann et al., 2020). Studies were categorized into the outcomes they measured. Outcomes were assessed using six criteria: study design, study limitations, inconsistency, indirectness, imprecision, and publication bias. Outcomes from RCTs and NRSIs were assessed separately.

### Data Synthesis and Meta-Analysis

A meta-analysis was conducted on trials measuring MUDR, as the trials included were homogeneous for outcomes. No other outcomes produced enough data to perform a meta-analysis. The PlotDigitizer software (Kadic et al., 2016) was used to manually extract the data from trials that did not report the mean difference or standard deviation (SD) for pre-and-post-MUDR for the control groups. The data from Del Vecchio et al. (2019a), was not possible to extract with this software from the graphs presented in the article; the author was contacted and provided the data to be used for meta-analysis. Vila-Chã et al. (2010) reported results for both the VL and VMO and these were inputted separately within the meta-analysis.

The I^2^ statistical analysis was used to evaluate the variation between studies that was due to heterogeneity rather than chance (Higgins et al., 2003). The Chi-squared test was also performed to further inform the potential heterogeneity present. Standardized mean difference (SMD) was calculated using Cohen’s D formula. The effect size was defined as small for effect size between 0.0 and 0.2, medium for effect size between 0.3 and 0.7 and large for effect size 0.8 or greater (Fritz et al., 2012). 95% Confidence Intervals (CI) was also calculated for MUDR (Altman et al., 2013). A random-effects meta-analysis model was used to account for heterogeneity caused by the variation of populations included in the trials and the varying dosage and length of RT (Riley et al., 2011). A sensitivity analyses was performed in case of high heterogeneity. All analyses were computed in Review Manager (RevMan) [Computer program]. Version 5.4, The Cochrane Collaboration, 2020. For all other variables, a narrative synthesis of results is presented.

## Results

### Study Identification and Characteristics

Initial searches using electronic databases resulted in 1,050 studies retrieved. After the removal of duplicates and screening of the titles and abstracts, the remaining 16 studies were subject to full-text review. A further nine studies were removed, leaving seven studies to be included for analysis (Figure 1). Study characteristics can be found for the seven included studies in Table 2. Five studies were RCTs (Rich and Cafarelli, 2000; Pucci et al., 2006; Vila-Chã et al., 2010; Stock and Thompson, 2014; Vila-Chã and Falla, 2016) and two studies were NRSIs (Del Vecchio et al., 2019a; Sterczala et al., 2020).

**TABLE 2:** Study characteristics.

Study (year)	Sample	Final sample size (dropouts) age (years mean ± SD)	Resistance training tasks(s) Characteristics Target Duration	EMG method Muscle(s) assessed	Outcome measure(s)
**Randomized control trials**					
Rich and Cafarelli, 2000	*N* = 20 males	Resistance *n* = 10 (0) Control *n* = 10 (0) Sample (22.9 ± 2.1 yr)	**Isometric resistance training.** Quadriceps of dominant leg. Five sets of 10 brief MVC (3–5 s) 24 sessions, 8 weeks	Intramuscular EMG Vastus lateralis	Motor unit discharge rate
Pucci et al., 2006	*N* = 20 males	Resistance *n* = 10 (0) Control *n* = 10 (0) Sample (25.0 ± 5.5 yr)	**Maximal Voluntary Contractions.** Quadriceps. Three sets of 10 MVCs (MVC was held for 3 s with 3 s intervals intervening) with 2 min rest between sets. 9 sessions, 3 weeks	Intramuscular EMG Right vastus lateralis	Motor unit discharge rate
Vila-Chã et al., 2010	*N* = 30 males	Resistance *n* = 9 (1) (25.4 ± 4.2 yr) Control *n* = 8 (2) 27.0 ± 5.0 yr Endurance *n* = 10 (0) (26.1 ± 2.8 yr)	**Variety of exercises.** Trunk, bilateral upper, and lower limbs. Three bilateral leg press, leg extension, and leg curl, and four additional lateral pull down, bench press, exercise for the trunk flexors and for trunk extensors. 18 sessions, 6 weeks	Intramuscular EMG Vastus lateralis and vastus medialis obliquus	Motor unit discharge rate
Stock and Thompson, 2014	*N* = 26 males	Resistance *n* = 15 (0) Control *n* = 9 (2) Aged 24 ± 3 years	**Conventional barbell deadlifts**. Knee extensors. Two warm-up sets of five repetitions. Three minutes of rest was allotted between each set. As a means of progressive overload, 0.45–2.2 kg was added to the barbell for each training session. 25 repetitions per sessions. 20 sessions, 10 weeks	Surface EMG array. Vastus lateralis	Mean firing rate vs. recruitment threshold relationship Firing Rate at recruitment vs. recruitment threshold relationship
Vila-Chã and Falla, 2016	*N* = 30 males	Resistance *n* = 9 (1) (25.4 ± 4.2 yr) Control *n* = 8 (2) (27.0 ± 5.0 yr) Endurance *n* = 10 (0) (26.1 ± 2.8 yr)	**Variety of exercises.** Trunk, bilateral upper, and lower limbs. Three bilateral leg press, leg extension, and leg curl, and four additional lateral pull down, bench press, exercise for the trunk flexors and for trunk extensors. 18 sessions, 6 weeks	Intramuscular EMG Vastus lateralis and vastus medialis obliquus	Motor unit discharge rate
**Non-randomized control trials**					
Del Vecchio et al., 2019a	*N* = 28 males	Resistance *n* = 13 (1) 23.9 ± 2.9 yr. Control *n* = 12 (2) 25.1 ± 2.9 yr.	**Ballistic and isometric contractions** Tibialis anterior Warm-up of (2 × 50, 2 × 70 1 × 90% of perceived MVF) followed by 3 MVCs then 40 maximal ballistic contractions (4 × 10 reps; 60 s with 1 min of recovery between sets), 4 min of rest, then 30 sustained isometric ramp contractions (3 × 10 reps; 60 s with 2 min of recovery). 12 sessions, 4 weeks (30 mins per session)	High density surface EMG Tibialis anterior	Motor unit discharge rate
Sterczala et al., 2020	*N* = 30 males	Resistance *n* = 16 (4) (20.7 ± 1.9 yr) Control *n* = 8 (2) (19.4 ± 2.5 yr)	**Variety of lower limb exercises based on linear periodization model.** Knee extensors. Back squats, front squats, Romanian deadlifts, knee extensions, leg presses, glute bridges, step ups, hamstring curls, and reverse hyperextensions. 3 sets of 12 repetitions during weeks 1–3, 3 sets of 8 repetitions during weeks 4–6 and 4 sets of 5 repetitions during weeks 7–8. 24 sessions, 8 weeks	5-pin surface EMG array. Vastus lateralis	Motor unit recruitment threshold Mean firing rate vs. recruitment threshold relationship

*EMG, electromyography; NR, Not reported.*

### Participants

A total of 167 participants were investigated across the seven studies, with a range of 20–27 participants. Ages ranged from 19.4 ± 2.5 to 27.0 ± 5.0 years. All studies investigated men only. Five studies separated participants into RT groups and control groups, whereas two studies separated participants into RT groups, endurance training groups and control groups (Vila-Chã et al., 2010; Vila-Chã and Falla, 2016).

### Study and Outcome Measure Characteristics

Further detailed study characteristics can be found in Supplementary Appendix 3. Four studies included MUDR as an outcome (Rich and Cafarelli, 2000; Pucci et al., 2006; Vila-Chã et al., 2010; Del Vecchio et al., 2019a), one study MUDRV (Vila-Chã and Falla, 2016) and two studies MURT (Del Vecchio et al., 2019a; Sterczala et al., 2020). Two additional outcomes are also presented: MUDR-vs.-RT relationship (Stock and Thompson, 2014; Sterczala et al., 2020) and MUDR at Recruitment vs.- RT Relationship (Stock and Thompson, 2014).

### Quality Assessment of the Included Studies

**FIGURE 2:**
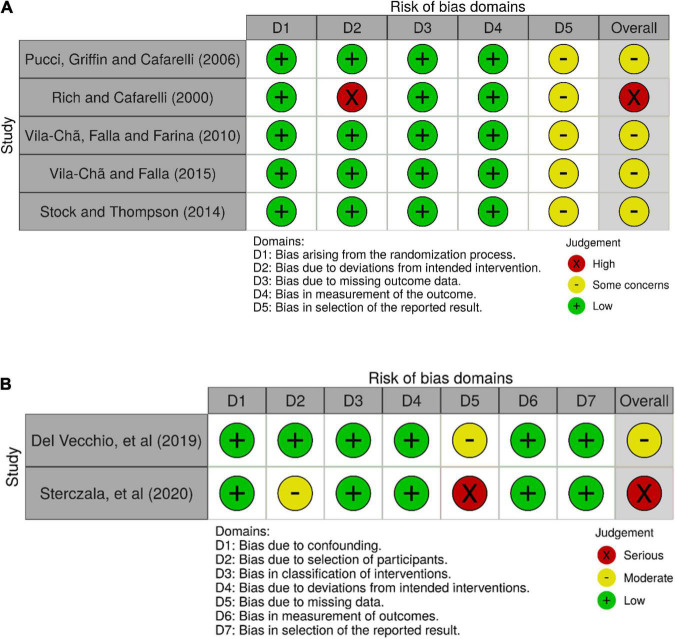
Risk of bias. **(A)** Results for RCTs, **(B)** results for NRSIs. *Created using Robvis: Mcguinness and Higgins (2021).

The ROB-2 tool was applied to five RCTs, and the ROBINS-I tool was applied to two NRSIs. Five trials were found to have an overall moderate risk of bias or presented some concerns, and the remaining two trials (Rich and Cafarelli, 2000; Sterczala et al., 2020) were identified as being at high risk of bias. The results for the ROB-2 assessments and the ROBINS-I assessments are visualized in Figures 2A,B, respectively. The assessment of the RCTs revealed some concerns in the bias in the selection of the reported results domain. This was a result of the potential unblinding of outcome data before statistical analysis was conducted (five studies). One study presented a high risk of bias due to deviations from intended interventions, within which, eight participants took a 1-week break from the RT program midway through the trial (Rich and Cafarelli, 2000).

The ROBINS-I tool assessment revealed that one of the NRSIs presented some concerns due to missing data, owing to an approximate 10% dropout rate (Del Vecchio et al., 2019a), and one study showed a high risk of bias due to missing data, as the study used ANOVA analysis despite the presence of missing data (approximate 20% dropout in control group; Sterczala et al., 2020).

### Confidence in Cumulative Evidence

The certainty of the evidence for each outcome assessed by RCTs and NRSIs was assessed separately by two reviewers (Schünemann et al., 2020) and is presented in Supplementary Appendixes 4, 5, respectively.

### Meta-Analysis

The number of studies measuring the outcome variables: MUDRV (*n* = 2), MURT (*n* = 2), mean discharge rate vs. recruitment threshold relationship (*n* = 2) and mean discharge rate at recruitment vs. recruitment threshold relationship (*n* = 1) were insufficient and produced limited data for pooling, consequently they were not considered for meta-analysis.

A meta-analysis was conducted on the outcome MUDR. The data from four trials that studied MUDR, including three RCTs (Rich and Cafarelli, 2000; Pucci et al., 2006; Vila-Chã et al., 2010) and one NRSI (Del Vecchio et al., 2019a) was pooled. These studies had 82 participants combined. Three out of four studies were found to have some concerns from the risk of bias assessment, and one study was identified as being at high risk of bias. This outcome was identified as having serious imprecision due to variation within the study designs (see section “Motor Unit Discharge Rate”).

As presented in Supplementary Appendix 6, the SMD was calculated at 0.70 toward the RT groups. Despite identifying this as a moderate effect size (Fritz et al., 2012), it is not statistically significant (*P* = 0.38). An I^2^ value of 91% was calculated, which when coupled with a significant Chi-squared test (*P* = 0.00001), means considerable heterogeneity is likely to be present. Due to the small number of studies in this meta-analysis this finding should be interpreted with caution. The sensitivity analyses revealed no significant difference after removal of the study with high risk of bias (*P* = 0.20), I^2^ value of 90% with a significant Chi-squared test (*P* = 0.00001).

### Synthesis of Results

#### Training Methods

Differences in training methods highlight a source of heterogeneity across the trials. Three trials used maximal isometric or isometric ballistic voluntary contractions to form the exercises making up their RT regimens (Rich and Cafarelli, 2000; Pucci et al., 2006; Del Vecchio et al., 2019a), whereas the other four studies used a combination of multiple lower body exercises including exercises that did not specifically isolate the muscles that were being used for measurement of motor unit firing properties; except for Stock and Thompson (2014) who used a RT program consisting of conventional deadlifts only. Vila-Chã et al. (2010), Vila-Chã and Falla (2016), and Sterczala et al. (2020), used a linear periodization model to guide the sets, repetitions and level of resistance used in the RT programs. Whereas, Rich and Cafarelli (2000), Pucci et al. (2006), and Del Vecchio et al. (2019a), opted for a non-adjustable and consistent program with multiple sets of maximal voluntary contractions (MVC) in each session. Additionally, there were differences in the limbs trained across studies. Rich and Cafarelli (2000) and Del Vecchio et al. (2019a), used a unilateral training method and Rich and Cafarelli (2000) specified that the dominant leg was trained. Whereas, the other trials used a bilateral training approach and Pucci et al. (2006), did not report this aspect of their training method. Details of the training methods used can be found in Supplementary Appendix 7.

#### Motor Unit Discharge Rate

The certainty of the evidence for MUDR was rated as very low. Three RCTs measured MUDR. Pucci et al. (2006), conducted a 3-week RT program (the shortest duration of all trials in this synthesis), measuring vastus lateralis (VL) *via* intramuscular EMG. They found no significant changes in MUDR at 50, 75, or 100% of MVC in the second or third week of the trial, respectively. Rich and Cafarelli (2000) conducted an 8-week RT program measuring the VL and found no difference in MUDR pre- and post-RT. Vila-Chã et al. (2010), measured both VL and vastus medialis obliquus (VMO) before, midway through, and immediately post a 6-week RT program. They found an initial significant increase in MUDR after 3 weeks (VMO only), and an increase of 1.28 ± 0.7 pps and 1.60 ± 0.8 pps in both VMO and VL after 6 weeks, respectively. Clinical and methodological heterogeneity is evident in the contrasting study design (Higgins et al., 2021). The duration of RT ranged from 3 to 8 weeks, involved different measurement points and not all studies measured MUDR midway through the trial. Further, clinical heterogeneity stems from differing participant groups. Additionally, serious imprecision was identified for this outcome; no sample size calculations are reported in any trials despite the total number of participants across these three studies being just 57. Combined with the serious limitation identified from the high risk of bias in one study (Rich and Cafarelli, 2000) this resulted in the certainty of evidence being downgraded to very low.

Only one NRSI studied MUDR (Del Vecchio et al., 2019a). In contrast with Pucci et al. (2006), who found no change after a 3-week RT program, Del Vecchio et al. (2019a) found a significant increase in MUDR after a similar 4-week RT program. Despite this, the overall certainty of the evidence for this outcome was identified as very low, owing to both to risk of bias due to missing data and serious imprecision due to small sample size and omission of appropriate sample size calculations (25 total participants). The included study also had uneven groups (13 in the RT group and 12 in the control group) and a large sample size effect despite no confidence intervals being reported.

#### Motor Unit Recruitment Threshold

Two NRSIs measured MURT (Del Vecchio et al., 2019a; Sterczala et al., 2020) with the results producing an overall very low certainty of evidence. Del Vecchio et al. (2019a) found a significant decrease in absolute MURT after 4-weeks of RT (*P* = 0.042). Whereas, Sterczala et al. (2020) reported a non-significant increase in mean ranges of MURT for the VL following an 8-week RT program (measured *via* 5-pin surface array EMG). Heterogeneity is present in the MURT outcome in the form of different training program timings and different muscles used for outcome measurements, which led to downgrading for serious indirectness. Unique to the MURT outcome, downgrading for serious inconsistency also occurred due to the contrasting direction and effect size of results. Serious imprecision was found for the MURT outcome due to the uneven splitting of RT and control groups. Additionally, Del Vecchio et al. (2019a) reported a large effect size without reporting confidence intervals further supporting the downgrading for serious imprecision in both outcomes. With such a small number of events for this outcome, a large treatment effect may be explained by prognostic imbalance (Guyatt et al., 2011).

#### Motor Unit Discharge Rate Variability

One RCT (Vila-Chã and Falla, 2016) measured MUDRV, reporting a significant (*P* = 0.001) reduction in MUDRV. This was graded as moderate certainty of evidence. Vila-Chã and Falla (2016), resulted in a borderline decision not to downgrade for study limitation due to a lack of clarity surrounding the unblinding of the results before statistical analysis was conducted. The decision to not downgrade was reached between the two reviewers, as this was unlikely to lower the confidence in the estimate of effect, thus not seriously limiting the trial. However, this outcome was downgraded due to serious imprecision due to the small sample size (10 participants in the RT group), an absence of sample size calculations, not reporting confidence intervals and uneven groups.

#### Motor Unit Discharge Rate vs. Recruitment Threshold Relationship

One RCT (Stock and Thompson, 2014) and one NRSI (Sterczala et al., 2020) measured this outcome. The RCT reported no change in the linear slope coefficients post-RT. However, the study only included 24 participants and reported no sample size calculations and was therefore downgraded for serious imprecision. This outcome was also borderline for being downgraded due to study limitations, owing to two participants being excluded before statistical analysis. However, the two reviewers agreed this was unlikely to lower the confidence in the estimate of the effect. The overall certainty of the evidence was rated as moderate.

Sterczala et al. (2020), also found no significant interactions between MUDR and MURT with no changes in y-intercepts being observed. According to GRADE, there is low certainty of the evidence for this outcome owing to serious imprecision, although upgrading for well-controlled confounders occurred.

Important methodological differences are present between the two studies measuring this outcome. Sterczala et al. (2020) conducted an 8-week lower-limb RT program based on a linear periodization model, whereas Stock and Thompson (2014), conducted a 10-week program of conventional deadlift RT. This methodological variation was due to Stock and Thompson (2014), aiming to investigate the effect of RT on motor unit behavior in large muscle groups such as RF, whilst also investigating these effects in more realistic and generalizable RT settings.

#### Motor Unit Discharge Rate at Recruitment vs. Recruitment Threshold Relationship

Only Stock and Thompson (2014) presented results for this outcome measure, reporting that the linear slope coefficient for the RT group was significantly less than the control group (20.15 vs. 20.04 pps/% MVC). The overall certainty of the evidence was rated as moderate due to downgrading *via* serious imprecision; this outcome is based on one study including 15 participants in the RT group, so lacks statistical power (Guyatt et al., 2011).

## Discussion

This is the first systematic review to evaluate the current evidence surrounding changes in motor unit firing properties following RT vs. a control group. This review found a moderate level of evidence supporting a decrease of MUDRV following RT and a very low certainty of evidence for no variation in MUDR (SMD = 0.7, *P* = 0.43), with a high likelihood of heterogeneity (*I*^2^ = 91%). Additionally, a low to moderate level of evidence was found for a change in the linear slope coefficient relationships between MUDR and MURT. None of the seven studies included in this review was rated as low risk of bias, therefore the outcomes should be interpreted with caution. More high-quality studies are needed before conclusions can be drawn. Despite this, important recommendations for future research can be drawn from this systematic review.

The studies included in this review had small sample sizes and lacked statistical power, not only reducing the chance of detecting a true effect but also making statistically significant results less likely to reflect a true effect (Button et al., 2013). Future studies should recruit larger sample sizes and report sample size calculations within their methods. Additionally, future research should strive to reduce imprecision. For example, the inclusion of women within future trials may account for observed sex differences in motor unit behavior during submaximal contractions (Peng et al., 2018; Inglis and Gabriel, 2020).

A large source of clinical heterogeneity within the current evidence comes from different RT regimens, as presented in Supplementary Appendix 7. Some trials used a constant RT regime, recording MVC to determine the intensity of contractions performed per session without altering these throughout the trial (Rich and Cafarelli, 2000; Pucci et al., 2006; Del Vecchio et al., 2019a) showing a variable strength increase. Other trials used a linear periodization system (Vila-Chã et al., 2010; Vila-Chã and Falla, 2016; Sterczala et al., 2020). A higher total volume of training occurs in the periodization model, an important factor for increasing strength (Ratamess et al., 2009). Increased intensity has also been shown to affect neural properties such as resting membrane potential and voltage threshold of α-motor neurons (Gardiner, 2006). Differences in single-joint vs. multi-joint exercises present an additional source of heterogeneity (Boccia et al., 2019). The current evidence on the physiological response to different training methods is thus inconclusive, which precludes a recommendation for future research (Schoenfeld et al., 2019).

There is much debate surrounding what causes an initial increase in strength before morphological changes occur in the muscular tissue. Although a combination of multiple neural factors are likely involved (Siddique et al., 2020; Pearcey et al., 2021; Škarabot et al., 2021; Tallent et al., 2021), it is not clear which factors are most relevant. A recent review concluded that the reticular formation and inhibitory cortical interneurons are involved in the neural response to RT (Škarabot et al., 2021). Furthermore, Siddique et al. (2020) found that the mediation of short-interval intracortical inhibition *via* GABA-ergic inhibitory circuits might also play a role in the increased efferent drive to agonists after RT, contributing to the early increases in strength. However, these findings illustrate subtle changes and are unlikely to be the only factors involved.

The present review has highlighted the potential of MUDR as a property that should be subject to further research. Despite low-quality trials and a high risk of bias, the significant increases in MUDR found by Vila-Chã et al. (2010) and Del Vecchio et al. (2019a) demonstrate there is potential for significant changes. Specifically, larger sample sizes, trials which incorporate a standardized RT program using a linear periodization model may produce significant results.

Another MU property highlighted by this review as likely to be changed by RT is MURT. Significant decreases in MURT were found in Del Vecchio et al. (2019a) after a 4-week RT program. These results seem to concur with a previous study which reported that MURT can initially decrease by as much as 40% in the first 24-h following eccentric exercises (Dartnall et al., 2011). The maintenance in MURT found by Sterczala et al. (2020), from a longer, 8-week program contradicts Del Vecchio’s results. This inconsistency is ultimately undermining the quality of the evidence, further reduced by the heterogeneity between studies. We hypothesize that the initial decrease in MURT observed by Dartnall et al. (2011), may continue for some weeks following the commencement of an RT program. However, a significant decrease cannot be observed at 8-weeks because of a gradual normalization of MURT back to pre-training levels, as muscle fiber changes begin to occur.

The different methods employed to identify motor units and assess their firing properties across the included studies are another important factor to consider. These different techniques may present some challenges since the examination of motor unit firing properties with methods with low-spatial resolution such as intramuscular EMG, may increase the likelihood of sampling different populations of motor units (i.e., low-vs. high-threshold motor units) across the training intervention. This is problematic since properties such as MUDR vary according to the MURT [i.e., low-threshold motor units usually show higher MUDR compared to high-threshold motor units (De Luca and Erim, 1994)]. Additionally, there is a need for a large number of motor units to be recorded to obtain reliable data for a pool of motor units with similar properties. Conversely, recordings with higher spatial resolution such as high-density surface EMG enable the assessment of similar populations of motor units across testing sessions (Martinez-Valdes et al., 2016) and in some cases (when changes in muscle volume are not too substantial) allow the tracking of the same motor units across the training intervention (Martinez-Valdes et al., 2017b) as recently shown by Martinez-Valdes et al. (2017a) and Del Vecchio et al. (2019b). Therefore, high-density EMG techniques might have the potential to further clarify the contribution of motor unit firing properties to the increase in strength observed after RT. However, their use in longer training interventions, inducing significant changes in muscle mass needs to be confirmed in future studies.

Other challenges when comparing studies assessing motor unit firing properties following a period of RT should be considered when interpreting the results of this systematic review. Important considerations are that different muscles have been examined, using different detection methods, and different training regimes have been employed. Some studies included in the review involved training with constant loads (requiring coordination between muscles), other used ballistic training, and some involved isometric training. These different training protocols most likely result in different changes in motor unit activity which impacts on drawing conclusions across multiple studies. Additionally, and as mentioned above, some studies use conventional intramuscular recordings, which is restricted to recordings during low force isometric contractions. In contrast, other studies used high-density EMG and a decomposition technique to extract motor unit activity from a large motor unit population during higher force contractions, but this is usually limited to more superficial motor units that are likely to be different to the motor units targeted with the conventional technique. Further, studies have obtained motor unit recordings from different muscles, which differ in the mode of action and the magnitude of coordination with other muscles. This may also contribute to different changes in motor unit activity with RT. Additionally, due to lack of evidence of the effects of RT in the same motor unit population (i.e., motor units tracked longitudinally across the period of training), it remains unclear whether the adaptations are uniform within the motor pool. All of the above impacts on the ability to draw conclusions from the current body of evidence and should be considered when interpreting the results of this review.

### Limitations

This review has systematically appraised the evidence regarding changes in some motor unit firing properties in response to RT. However, limitations of the review must be acknowledged. Although a search of multiple databases was performed, the literature search was limited to online databases. The pre-established eligibility criteria determined the exclusion of prospective cohort studies without comparison to a control group that did not receive the RT intervention. This strict criterion limited the inclusion of some publications that are considered seminal work in area (e.g., Van Cutsem et al., 1998). Gray literature was not initially searched, although it was later searched during the GRADE assessment, and no publication bias was identified. Almost all trials included had some missing data, reflected in the risk of bias assessment. One study author was contacted in a request for non-reported MUDR data. Non-reported data from other trials studying MUDR was manually extracted using Plot Digitizer software to complete the meta-analysis. Despite this software having higher interrater reliability than manual extraction (Kadic et al., 2016), the data was not extracted by two separate reviewers, increasing the risk of measurement bias. Also, relevant motor unit properties such as motor unit conduction velocity (MUCV), were not included in this review. For example, Casolo et al. (2020) found that high threshold motor units show specific adaptations to isometric RT, likely due to changes in muscle membrane properties. However, the current number of studies investigating the effects of RT on MUCV is low, and a high certainty of evidence was unlikely to have been found. A further consideration is that we did not include an upper limit of the duration of training in the search strategy. Yet there is a general consensus that neural changes to RT are most notable with short-term resistance training, with morphological changes being the primary mechanism for long-term strength gains.

## Conclusion

There is a lack of high-quality evidence to demonstrate the effect of RT on motor unit firing properties. The meta-analysis showed that there is no change in MUDR following RT although this should be interpreted with caution given the heterogeneity across studies particularly in relation to the muscle examined, the detection method used to assess MU activity and the type of RT used. A narrative synthesis revealed a mixed direction of evidence for most outcomes, with very low-to-moderate certainty of evidence. Clinical heterogeneity lies throughout this body of research, methodological differences between studies undermine the certainty of evidence, and differences between types of RT may have an impact on motor unit behavior.

## Data Availability Statement

The original contributions presented in the study are included in the article/Supplementary Material, further inquiries can be directed to the corresponding author.

## Author Contributions

DF, EE-C, and EE conceived and designed research. EE first reviewed, EE-C second reviewed, and DF third reviewed the manuscript. EE and EC drafted the manuscript. EE, EE-C, EM-V, and DF revised the manuscript and approved final version of the manuscript. All authors contributed to the article and approved the submitted version.

## Conflict of Interest

The authors declare that the research was conducted in the absence of any commercial or financial relationships that could be construed as a potential conflict of interest.

## Publisher’s Note

All claims expressed in this article are solely those of the authors and do not necessarily represent those of their affiliated organizations, or those of the publisher, the editors and the reviewers. Any product that may be evaluated in this article, or claim that may be made by its manufacturer, is not guaranteed or endorsed by the publisher.
