# Supplementary material for: The Effect of Resistance Training on Motor Unit Firing Properties: A Systematic Review and Meta-Analysis
Source: Front Physiol. 2022 Feb 28;13:817631. doi: 10.3389/fphys.2022.817631 (PMC8918924; doi:10.3389/fphys.2022.817631)
Supplement: Supplementary file 1 [file Data_Sheet_1.docx]

**SUPPLEMENTAL ONLINE MATERIAL**

**Appendix 1:** Search String Example

**Database:** PubMed

**Search Date:** 25/06/21

**Search Specification:** All search terms were in MeSH where possible. A publication year filter was applied at 1964- search date.

**Results**: 18,410

| "motor neurons"[Mesh:NoExp] OR “motor unit*”[tw] OR “motor neuron” [tw] OR “motor output” [tw] OR “motor activity” [tw] OR “neural changes” [tw] OR “neural adaptation*”[tw] OR “neuromuscular adaptation” [tw] OR “neuromuscular changes” [tw] OR "resistance training"[Mesh] OR “resistance exercise” [tw] OR “resistive exercise” [tw] OR “strength exercise” [tw] OR “strength training” [tw] OR “strengthening” [tw] OR “resistive training” [tw] OR "Electromyography"[Mesh] OR “EMG” [tw] OR “discharge rate variability” [tw] OR “inter-spike interval” [tw] OR “inter-spike interval variability” [tw] OR “force steadiness” [tw] OR “discharge rate” [tw] OR “firing rate” [tw] OR “firing frequency” [tw] OR “recruitment threshold” [tw] OR “motor unit recruitment” [tw] OR “motor unit behaviour” [tw] |
| --- |

**Appendix 2:** Search Terms

**Appendix 3:** Detailed Study Characteristics

| Study (year) | Maximal Voluntary contraction  (MVC)  Percentage strength change(%) | Motor Units Recorded | Motor Unit Discharge Rate  (MUDR) | Motor Unit Discharge Rate Variability  (MUDRV) | Motor Unit Recruitment Threshold  (MURT) | Motor Unit Derecruitment Threshold | Other | Differences Between Groups |
| --- | --- | --- | --- | --- | --- | --- | --- | --- |
| Del Vecchio et al (2019) | **Resistance Training:** Pre: 289 ± 64 N, Post: 329 ± 61 N (14%)  **Control:** Pre: 299 ± 41 N, Post: 304 ± 35 N | Total: 2560 | **Resistance Training:** Average increase across forces: 3.3 ± 2.5 pps. **Control:** NR. | NR | **Resistance Training:** Absolute Recruitment Pre: 77 ± 24 Post: 71 ± 23 (interaction: time × group; P = 0.042, ηp² = 0.131).  **Control:** NR.  Relative recruitment: Resistance Training: Pre: 26 ± 5 Post: 21 ± 5 (MVC). **Control:** NR. | **Resistance Training:** Pre: 79 ± 25 Post: 97 ± 31 (interaction: time × group; P = 0.017, ηp² = 0.225).  **Control:** NR. |  | **MUDR:** Resistance Training: increase at 35% MVC: main effect for time; p = 0.015, ηp2 = 0.402. 50%: P = 0.009, ηp2 = 0.450. 70%: P = 0.001, ηp2 = 0.599. |
| Pucci et al (2006) | **Resistance Training:**  Day 1: 761+77 N. Day 5: 132 ± 19 mV. Day 9: 1031 + 78 N (35%). **Control:** Day 1: 848 + 77N , post: 835.1 + 70 N | **Resistance Training:** 340  **Control:** 191 | **Resistance Training:** day 1: 50% MVC: 15.51 ± 1.48 Hz  75%: 20.23 ± 1.85,  100%: 42.25 ± 2.72. Day 5: 50% MVC: 14.71 ± 1.18,  75%: 20.61 ± 1.37,  100%: 40.18 ± 4.14. Day 9: 50%: 16.23 ± 1.56,  75%: 21.57 ± 1.84,  100%: 43.46 ± 2.63.  **Control:** NR. | NR | NR | NR |  | **MUDR**: no change  50% MVC (15.51 ± 1.48 Hz)  75%MVC (20.23 ± 1.85 Hz)  100% MVC (42.25 ± 2.72 Hz) |
| Rich et al (2000) | **Resistance Training MVC:** Pre: 541.06 ± 35.44 N Post: 736.50 ±74.98. (36%) TwMax Pre: 104.7± 11.36 N, Post: 122.9±13.47 N.  **Control: TwMax** Pre: 115.4 ±11.31 N, Post: 98.8±9.47 N. | NR | **Resistance Training:** Pre: 12.46 ± 1.25 Hz, Post: NR.  **Control:** Pre: 11.41 ± 0.19. Post: NR | NR | NR | NR |  | **MVC:** 36% increase (P <0.05).  **Average MUDR:** no difference. |
| Vila-Chã et al (2010) | NR (17.5 + 7.5%) | NR | NR | NR | NR | NR |  | **MVC: Resistance Training:** MVC increased 17.5 ± 7.5%. **Rate of force development** increased 33.3 ± 15.9%.  **Control:** No change in any motor unit parameters.  **Endurance:** non-significant decrease in MVC after 3 weeks.  **Average motor unit discharge time:**  **Resistance Training:** after 3 weeks increased (in VMO) 8.4 ± 5.2% (for VMO p< 0.05; VL, p=0.12).  6 weeks: average MUDR increased by 10.7 ±4.9% (VMO) and 12.8 ± 4.7% (VL). MUDR VMO increased 1.28 ± 0.7 pps, VL increased by 1.60 ± 0.8 pps. |
| Vila-Chã et al (2016) | **Resistance Training:** Pre: 532.1 ± 107.9 N, Post: 622.7 ± 118.6 N (P < 0.05). (17.5 ± 7.5%)  **Control:** Pre: 549.5 ± 68.5 N, Post: 554.4 ± 47.2 N.  **Endurance:** Pre: 31.3 ± 95.3 N  Post: 507.7 ± 126.5 N. | Total: 1017 | NR | **VMO**  **Resistance Training:** Pre: 13.3, Post: 12.7.  **Control:** Pre:13.8, Post:13.9  **Endurance:** Pre: 15.3, Post: 11.1.  **VL**  **Resistance Training:** Pre: 11.6, Post: 13.6.  **Control:** Pre: 11.9, Post: 11.3.  **Endurance:** Pre: 12.4, Post: 11.7. | NR | NR |  | **Force steadiness** improved with Resistance Training but not with endurance training (group × time: P < 0.05), coefficient of variation of force decreased 27.2 ± 17.1% overall (P < 0.01). No change in endurance training group or control group.  **MVC** did not increase in the endurance or control group.  **MUDRV:** no significant change with different loads (P = 0.33). 20% MVC was higher than at 30% (13.1 ± 3.5% vs 12.4 ± 2.9%, respectively). MUDRV was significantly reduced for VMO and VL post resistance training (P = 0.001), did not change in the endurance or control group ( P = 0.875 and P = 0.995, respectively). |
| Sterczala et al (2020) | **Resistance Training:**  Pre: 204.6 ± 34.9 Nm, Post: 239.8 ± 36.3 Nm (p < 0.001, d = 0.9) (17%) **Control:** Pre: 182.4 ± 42.0 Nm, Post: 190.9 ± 40.1 Nm (p = 0.079; d = 0.2). | Total: 825  **Resistance Training:**  Pre: 16.3 ± 3.7, Post: 16.8 ± 6.0.  **Control:** Pre: 18.4 ± 4.4, Post: 18.5 ± 5.9. | NR | NA | **Resistance Training:** Pre: 25.1–61.9%, Post: 30.2–62.6%  **Control:** Pre: 22.4–60.7%, Post: 25.6–59.0%. | NR |  | **Mean firing rate versus recruitment threshold relationship:** no significant two-way interactions (p = 0.838, p = 0.949).  **Resistance training** larger MUAP-AMPS than the control group post- training (p = 0.045, d = 0.9).  **Mean Firing rates**: no change between observed motor units (two-way interaction: p = 0.864; time: p = 0.619; group: p = 0.869). |
| Stock et al (2014) | NR | **Pre-test:** VL:28 RF:28.  **Post-test (pre-test force):** VL:24 RF:28  **Post-test (new force):** VL:24 RF 31. | NR | NR | NR | NR | **Mean firing rate versus recruitment threshold relationship:** VL: NR, RF: NR.  **Firing rate at recruitment versus recruitment threshold relationship** (50% pre-test MVC): VL: Pre: 11.4pps, Post: 9.6pps. RF: NR. | **MVC:** increased in Resistance Training (p = .002, Cohen's d = .70), but not in control group (p = .992, Cohen's d<.01).  **Force steadiness:** two-way mixed factorial ANOVA no interaction (p = .580, ή2 = .024) and no main effect for force level (p = .096, ή2 = .101) or group (p = .434, ή2 = .028).  **Mean firing rate versus recruitment threshold relationship:** linear slope coefficients: no interaction (p=.132, n2 .088), and no main effect for force level (p=.091, n2=.103) or group (p=.397, n2=.033). Y- intercepts: no interaction (p=.627, n2=.021) and no main effect for group (p=.797, n2=.003).  **Firing Rate at Recruitment versus Recruitment Threshold Relationship:** RF main effect for group (p=.046, n2.170).  Linear slope coefficients: Resistance Training significantly less than control group (20.148 versus 20.038 pps/% MVC). The 95% CI for mean difference is 20.217 to 0.002 pps/% MVC (p=.046). |

*VL: vastus lateralis, VMO: vastus Medialis Obliquus, RF: rectus femoris, NR: Not reported.

**Appendix 4:** GRADE Assessment for RCTs

| **No. of Studies (total participants)** | **Design** | **Study Limitation** | **Inconsistency** | **Indirectness** | **Imprecision** | **Publication Bias** | **Certainty**  **(overall score)** |
| --- | --- | --- | --- | --- | --- | --- | --- |
| **Outcome:** Motor Unit Discharge rate (MUDR) | | | | | | | |
| Pucci et al (2006), Rich et al (2000), Vila-Chã et al (2010)  (57) | Randomized control trials | Downgraded one level for serious limitation: all three studies measuring motor unit discharge rate had some concerns due to the unblinding of results being unclear. Rich, et al (2000) also had a high risk of bias due to deviation from the intended intervention but had the joint smallest number of participants (10). | No downgrading | Downgraded one level for serious indirectness: All trials measure MUDR directly. All trials study the effects of a similar resistance training programme. However, duration of the training ranged from 3 to 8 weeks. Vila-Cha, et al (2010) and Pucci, et al (2006) included extra measurements midway through the trials. All studies included adult males aged between 18-65. All studies compare RT to a control group (except Vila-Cha, et al (2010), which also compared to an endurance training group) which maintained normal physical activity. | Downgraded one level for serious imprecision: The total number of participants across studies for this outcome (excluding dropouts) was 57. All studies reported either no effect or none-significant results, possibly due to a small number of participants in each study. No sample size calculations were reported in any studies, and none were found from similar (non-included) studies in the literature. | None detected | 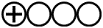 **Very low** |
| **Outcome:** Motor Unit Recruitment Threshold (MURT) | | | | | | | |
| No randomized control trials measuring this outcome. | NA | NA | NA | NA | NA | None detected | NA |
| **Outcome:** Motor Unit Derecruitment Threshold (MUDT) | | | | | | | |
| No randomized control trials measuring this outcome. | NA | NA | NA | NA | NA | None detected | NA |
| **Outcome:** Motor Unit Discharge Rate Variability (MUDRV) | | | | | | | |
| Vila-Chã et al (2016)  (17) | Randomized control trial | Not Downgraded (borderline)** | Only one study meaning inconsistency is not applicable. | Not Downgraded. | Downgraded one level due to serious imprecision: The evidence for this outcome is based on one study which reported reduction in MUDRV (P = 0.001). However, this study only included 10 participants in the RT group, which may impact the statistical power of results, and does not negate the low number of participants. | None detected | 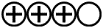 **Moderate** |
| **Outcome:** Motor Unit Discharge Rate versus Recruitment Threshold Relationship | | | | | | | |
| Stock et al (2014) (24) | Randomized control trial | Not downgraded but borderline* | Only one study meaning inconsistency is not applicable. | Not Downgraded | Downgraded one level for serious imprecision: This outcome is based on just one study which reported no effect. This study included 15 participants in the RT group, which may impact the statistical power of results. | None detected | 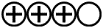 **Moderate** |
| **Outcome:** Motor Unit Discharge Rate at Recruitment versus recruitment Threshold Relationship | | | | | | | |
| Stock et al (2014) (24) | Randomized control trial | Not downgraded but borderline* | Only one study meaning inconsistency is not applicable. | Not downgraded | Downgraded one level for serious imprecision: This outcome is based on just one study which reported no effect. This study included 15 participants in the RT group, which may impact the statistical power of results. | None detected | 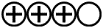 **Moderate** |

**Appendix 5:** GRADE assessment for NRSIs

| **No. of Studies (total participants)** | **Design** | **Study Limitations** | | **Inconsistency** | **Indirectness** | | **Imprecision** | | **Publication Bias** | **Certainty**  **(overall score)** |
| --- | --- | --- | --- | --- | --- | --- | --- | --- | --- | --- |
| **Outcome:** Motor Unit Discharge Rate (MUDR) | | | | | | | | | | |
| Del Vecchio et al (2019)  (25) | Non-randomized control trials. | **Downgraded** for limitations in design, bias due to missing data. | | Only one study meaning it is not possible to judge for inconsistency.  **Do not downgrade** | No indirectness | | **Serious imprecision:** Small sample size, no sample size calculation, the groups were uneven (13/12), no CI reported but large sample size effects. | | None detected | 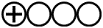 **Very low** |
| **Outcome:** Motor Unit Recruitment Threshold (MURT) | | | | | | | | | | |
| Del Vecchio et al (2019),  Sterczala et al (2020)  (49) | Non-randomized control trials. | **Downgraded** for limitations in design | | **Direction and magnitude of results across studies** were different while Del Vecchio showed decrease, and Sterczala did not change. | **Differences in interventions:**  Del Vecchio conducts a 4-week strengthening training regimen whereas Sterczala conducts an 8-week regimen. | | **Serious imprecision:** Small sample size, no sample size calculation, the groups were uneven Del Vecchio (13/12), Sterczala (16/8) no CI reported. but large sample size effects | | None detected | 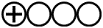 **Very low** |
| **Outcome:** Motor Unit Derecruitment Threshold | | | | | | | | | | |
| Del Vecchio et al (2019)  (25) | Non-randomized control trials | **Downgraded** for limitations in design | | Only one study meaning it is not possible to judge for inconsistency.  **Do not downgrade** | No indirectness | | **Serious imprecision:** Small sample size, no sample size calculation, the groups were uneven (13/12), no CI reported but large sample size effects. | | None detected | 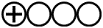 **Very low** |
| **Outcome:** Motor Unit mean Discharge Rate versus Recruitment Threshold Relationship | | | | | | | | | | |
| Sterczala et al (2020) (24) | Non-randomized control trials | **Upgraded** for when confounding is expected to reduce an effect: Sterczala (2020) excluded two participants for excess subcutaneous adiposity, potentially reducing the effect of a confounder, adiposity. | Only one study meaning it is not possible to judge for inconsistency.  **Do not downgrade** | | | No indirectness | | **Serious imprecision:** Small sample size, no sample size calculation, the groups were uneven (16/8) no CI reported. | None detected | 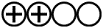 **Low** |
| **Outcome:** Motor Unit mean Discharge Rate at Recruitment versus Recruitment Threshold Relationship | | | | | | | | | | |
| No non-randomized studies measuring this outcome | NA | NA | NA | | | NA | | NA | NA | NA |

**NA: Not applicable.*

**Appendix 6:** Meta-analysis for changes in motor unit discharge rate following resistance training (RT) versus control


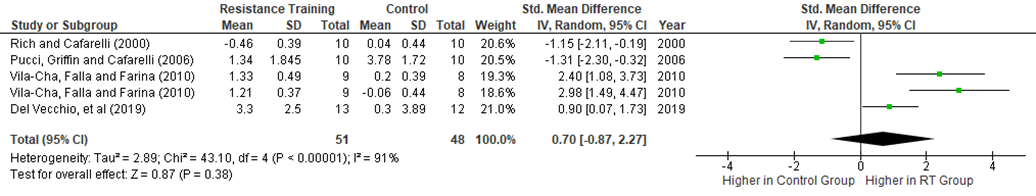


**Appendix 7:** Training Methods

| Study | Unilateral/ Bilateral | Training Type | Sets x Repetitions (reps) | Sessions/ Weeks | Linear Periodization Utilized? | Exercises Based on MVC? |
| --- | --- | --- | --- | --- | --- | --- |
| Del Vecchio et al (2019) | Unilateral (dominant leg) | Maximal ballistic contractions. | 4x10 reps | 12 sessions/ 4 weeks | No | Yes |
| Pucci et al (2006) | NR | Maximal voluntary contractions. | 3x 10 reps | 9 sessions/ 3 weeks | No | Yes |
| Rich et al (2000) | Unilateral (dominant leg) | Maximal Voluntary Isometric contractions. | 5x 10 reps | 24 sessions/ 8 weeks | No | Yes |
| Vila-Chã et al (2010) | Bilateral | Multiple lower limb exercises and exercises targeting other major muscle groups. | Varied based on linear periodization. | 18 sessions/ 6 weeks | Yes | No |
| Vila-Chã et al (2016) | Bilateral | Multiple lower limb exercises and exercises targeting other major muscle groups. | Varied based on linear periodization. | 18 sessions/ 6 weeks | Yes | No |
| Stock et al (2014) | Bilateral | Conventional Deadlifts | 5x 5 reps, or 6 sets if required to reach 25 total reps. | 20 sessions/ 10 weeks | No | No |
| Sterczala et al (2020) | Bilateral | Lower- body RT program- multiple exercises. | Varied based on linear periodization. | 24 sessions/ 8 weeks | Yes | No |
